# How zebrafish research has helped in understanding thyroid diseases

**DOI:** 10.12688/f1000research.12142.1

**Published:** 2017-12-14

**Authors:** Federica Marelli, Luca Persani

**Affiliations:** 1 Department of Clinical Sciences and Community Health, University of Milan, Milan, Italy; 2 Lab of Endocrine and Metabolic Research, IRCCS Istituto Auxologico Italiano, Milan, Italy

**Keywords:** zebrafish, thyroid

## Abstract

Next-generation sequencing technologies have revolutionized the identification of disease-causing genes, accelerating the discovery of new mutations and new candidate genes for thyroid diseases. To face this flow of novel genetic information, it is important to have suitable animal models to study the mechanisms regulating thyroid development and thyroid hormone availability and activity. Zebrafish ( *Danio rerio*), with its rapid external embryonic development, has been extensively used in developmental biology. To date, almost all of the components of the zebrafish thyroid axis have been characterized and are structurally and functionally comparable with those of higher vertebrates. The availability of transgenic fluorescent zebrafish lines allows the real-time analysis of thyroid organogenesis and its alterations. Transient morpholino-knockdown is a solution to silence the expression of a gene of interest and promptly obtain insights on its contribution during the development of the zebrafish thyroid axis. The recently available tools for targeted stable gene knockout have further increased the value of zebrafish to the study of thyroid disease. All of the reported zebrafish models can also be used to screen small compounds and to test new drugs and may allow the establishment of experimental proof of concept to plan subsequent clinical trials.

## Introduction

Thyroid hormones (THs) play an essential role in vertebrate development and metabolism regulation. The thyroid gland is then principally controlled by the negative feedback exerted by circulating THs at the pituitary and hypothalamic level in order to gauge the secretion of TSH, the thyroid-stimulating hormone ^1^. Thyroid function is further controlled at the tissue level by the peripheral gatekeepers of TH action, including the TH transporters (e.g. MCT8) and the iodothyronine deiodinases. In most species, deiodinases type 1 and 2 enzymatically convert the pro-hormone thyroxine (T4) to the bioactive hormone trio-iodothyronine (T3) and deiodinases type 1 and 3 can instead inactivate T3 to di-iodothyronine, T2. Ultimately, binding of T3 to its base on nuclear receptors (TRs) regulates the expression of specific genes by activating or suppressing transcription rates. Thus, proper TH action requires 1) the development of a functional hypothalamic-pituitary-thyroid axis, 2) transport of THs across the cell membrane, 3) an appropriate TH intracellular metabolism, and 4) T3 binding to the nuclear TRs and activation of gene expression ^2^. Thus, mutations on genes involved in all of these steps result in a wide range of TH defects (e.g. congenital hypothyroidism [CH], TH cell membrane transporter defect [THCMTD], or resistance to TH [RTH]) ^2– 4^. Although the pathological mechanisms can be very different, all of them result in aberrant expression of the T3 target genes required for the development and function of organs and tissues. As a result, TH defects not only delay normal development but also lead, if untreated, to irreversible lifelong defects in the nervous system. Cutting-edge technologies, such as those based on next-generation sequencing, are continuously providing unprecedented insights into the mechanisms involved in several forms of congenital thyroid defects. The challenge that we now have to face is identifying powerful systems for the functional validation of new hypotheses or putative pathogenic variants in candidate genes. *In vitro* assays or mouse models certainly continue to play a vital role in such functional validation strategies, but in recent years the zebrafish has also become a relevant model to test novel hypotheses or understand the role of candidate genes for several human diseases. Accordingly, this model has been more and more frequently used in studies on thyroid pathophysiology because of its versatility, limited costs, and the relatively shortened time to obtain significant results when compared with other animal models as well as the broad conservation of the molecular mechanisms involved in thyroid development and function and TH action ^5, 6^.

**Figure 1.**
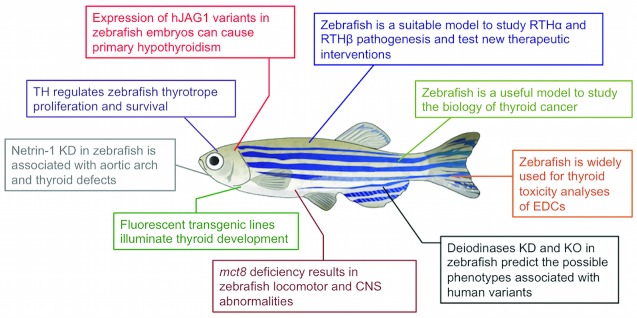
Schematic illustration of the most recent findings concerning thyroid development and thyroid hormone (TH) action that have been obtained using zebrafish as a model system. CNS, central nervous system; EDC, endocrine disruptor chemical; KD, knockdown; KO, knockout; mct8, monocarboxylate transporter 8; RTH, resistance to thyroid hormone

Here, we review the current knowledge on the development of the thyroid system in zebrafish, how recent clinical observations on this model shaped our understanding of thyroid diseases, and how these insights might be translated into therapeutic approaches ( Figure 1).

## Zebrafish to study thyroid development

In humans, CH represents the most common congenital endocrine disorder, affecting 3–6 out of 10,000 neonates. It is classically described to be a sporadic disease, but recent data support a possible oligogenic origin of CH, whose pathogenesis could thus frequently depend upon the combination of hypomorphic alleles of developmental or functional genes ^3^. However, the molecular mechanisms underlying CH, and thyroid dysgenesis in particular, remain largely unexplained. This is, in part, due to our still-limited understanding on how the function of intrinsic factors (e.g. thyroid-specific transcription factors) and extrinsic signaling cues (e.g. morphogens and growth factors) are integrated to regulate thyroid organogenesis.

The study of thyroid anatomy and its alterations after genetic manipulation using the zebrafish model offers several advantages that could overcome some of the technical limitations that are associated with using mammalian models. In contrast to intrauterine development in mammals, zebrafish embryos and larvae develop externally and are optically transparent, making them accessible to experimental manipulation and real-time observations throughout their entire embryonic development. These properties coupled with the availability of transgenic embryos expressing fluorescent reporter proteins in specific cell types allow the visualization and the study of organ development, including that of the thyroid gland. Opitz and colleagues have developed a panel of transgenic zebrafish lines in which the promoter of thyroglobulin ( *tg*) was used to drive the expression of different fluorescent proteins that allow time-lapse live imaging and real-time visualization of the dynamic changes in size, shape, and location of the developing zebrafish thyroid ^5, 7^.

Moreover, the availability of tools for the knockdown (morpholinos [MOs]) or knockout (e.g. the CRISPR/Cas9 system) of a gene of interest facilitates the analysis of the impact of a certain mutation during thyroid development. In modeling diseases using zebrafish, choosing between MOs and the CRISPR/Cas9 system depends on the particular situation. MOs are an established tool that has been utilized for the functional knockdown of many genes, while the CRIPSR/Cas9 system is an integrated toolbox that facilitates functional gene perturbations, such as loss and gain of function ^8^. MOs are designed to investigate phenotypes in the early developmental stages of the fish; however, the normal point at which to observe phenotypes in a CRISPR-Cas9-mediated transgenic fish is following the generation of F2, which normally takes over six months. Interestingly, a discrepancy was reported in the phenotypes between the previously described MO-treated fish and recently generated transgenic fish ^9^. The use of MOs is discouraged in some cases, considering the unprecedented phenotypes and possible off-target effects. However, in other cases, MOs work extremely well and efficiently mimic mutant phenotypes observed either in humans or in experimental models. Surprisingly, complete gene knockout was reported to be associated with the absence of the expected phenotype because of the unpredictable induction of a genetic compensation by other related genes able to offset the phenotype seen with MO ^10^. Interestingly, the compensation does not happen with MO. Thus, both MO- and CRISPR/Cas9-based approaches can be valuable and their application depends upon the aims of the study.

**Figure 2.**
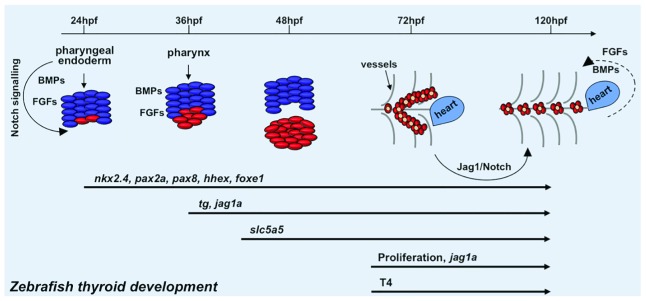
Schematic illustration of zebrafish thyroid development (modified from 7). The thyroid primordium rises from the pharyngeal endoderm around 24 hours post-fertilization (hpf) and expresses the early thyroid genes *nkx2.4*, *pax2a*, *pax8*, *hhex*, and *foxe1*. The differentiation of thyroid precursors continues up to 48hpf, at which they begin to express the late thyroid genes *tg* and *slc5a5*. The development of the zebrafish thyroid gland is complete at 72hpf. From this time, the thyroid follicles proliferate and localize along the dorsal aorta and start to produce T4. Intrinsic and extrinsic factors (Jag1-Notch, bone morphogenetic protein [BMP], and fibroblast growth factor [FGF] signaling) are also reported to play a pivotal role during thyroid organogenesis. For a detailed description of the molecular mechanisms underlying zebrafish thyroid morphogenesis, we refer the reader to the review by Fagman and Nilsson ^11^.

Despite the anatomic differences ( Figure 2), the molecular mechanisms of thyroid organogenesis appear to be well conserved between zebrafish and mammalian models. For a detailed description of zebrafish thyroid organogenesis and the underlying molecular events, the readers are referred to an excellent review ^11^. During the first three days of development, the zebrafish thyroid gland is not yet formed and the embryos are dependent on the maternal THs stored in the yolk sac ^5, 12^. As development progresses, the thyroid is formed from a midline anlage in the pharyngeal floor consisting of foregut endoderm cells that are committed to a thyroid fate. These thyroid progenitors give rise specifically to the follicular cells that proliferate to form mature TH-producing follicles. As described in mice, knockdown experiments in zebrafish demonstrated that the transcription factors nkx2.1, pax2a, and hhex are necessary for the specification and differentiation of thyroid primordium ^11^. The only exception is foxe1, which regulates thyroid primordium migration in higher vertebrates and mimics the picture of thyroid ectopia in nearly 50% of mutant mice ^11^. In zebrafish, foxe1 is expressed in thyroid progenitors, but no effects on thyroid morphogenesis were observed in foxe1 MO-injected zebrafish embryos ^13^. These authors argued that the zebrafish thyroid develops very early during development and foxe1’s role might be redundant in this context; it is thus possible that the role of *FOXE1* in mammalian thyroid development may have been acquired during evolution.

Once the thyroid gland is developed, it starts to produce THs, whose levels are finely regulated by the hypothalamic–pituitary–thyroid axis. Tonyushkina *et al.* developed a transgenic zebrafish that expresses GFP under the control of the *tshba* promoter (orthologous to the human TSH). They observed that the TH-driven negative feedback regulation of *tshba* transcription appears by four days post fertilization, the time in which the thyroid gland is already active ^14^. Interestingly, the authors showed that early exposure to elevated or low TH levels leads to thyrotrope cell death or hyperplasia, respectively ^14, 15^. These data demonstrate that transient TH changes profoundly impact the thyrotrope population during a critical period of pituitary development and may have long-term implications for the TSH set point later in life.

We have recently used the zebrafish model to analyze the functional consequences of several human JAG1 variants. JAG1 is a ligand of the Notch receptors that has been found to be mutated in patients with CH ^16^. We observed that knockdown of the zebrafish orthologues *jag1* and *ja1b* results in thyroid hypoplasia and TSH elevation that can be normalized by the human wild-type JAG1. Confirming the results observed *in vitro*, co-injection with the human JAG1 variants in zebrafish morphants failed to revert, at various degrees, the associated defects ^16^, emphasizing the usefulness of the zebrafish model to study the contribution of JAG1/Notch to thyroid function.

Pappalardo and colleagues used the zebrafish to study the role of Taz, a signal-responsive transcriptional coregulator, in thyroid formation ^17^. The *wwtr1* (encoding for the zebrafish Taz) transcript is expressed in the thyroid primordium and pharyngeal tissue of developing zebrafish, but its knockdown has only little effects on the expression of thyroid transcription factors and differentially alters the expression of thyroid differentiation genes. In later stages, Taz-depleted larvae exhibited a reduced number of thyroid follicle cells, indicating that the zebrafish Taz protein is required for normal thyroid morphogenesis ^17^. These data point to considerable genetic heterogeneity in this disorder, although no genetic alteration has been described in the *TAZ* gene in patients with CH so far.

To study CH pathogenesis comprehensively, the influence of the surrounding tissues during thyroid development should be taken into account. Opitz *et al.* recently described the role of netrin-1 in the pathogenesis of CH. Netrin-1 mutations have been detected in patients with co-occurring thyroid dysgenesis and congenital heart disease ^18^. In zebrafish, *ntn1a* is expressed in the pharyngeal arch and mesenchyme but not in the thyroid tissue. *Ntn1a*-deficient embryos displayed defective development of the aortic arch artery and abnormal thyroid morphogenesis. The thyroid phenotype is a result of the lack of proper guidance exerted by the dysplastic vasculature ^18^. Considering the close spatiotemporal relationship between thyroid and outflow tract development and the strong influence of the signals emanating from the cardiac mesoderm on thyroid development, such as BMPs and FGFs, netrin-1 is a strong candidate for having a direct role during thyroid development.

## Zebrafish to study thyroid cancer

A couple of recent manuscripts described the use of zebrafish to study the biology of thyroid cancer. Our group reported that patient-derived xenografts from papillary thyroid cancer or normal tissue stimulate the migration and the growth of sprouting vessels towards the implant in zebrafish embryos. This *in vivo* model could thus be considered as a valuable platform to test the effects of anticancer drugs ^19^. More recently, a novel model of thyroid carcinoma in zebrafish was reported ^20^. Through the use of real-time *in vivo* imaging, the authors studied a zebrafish line expressing BRAFV600E in thyrocytes that developed invasive carcinoma occurring early in thyroid development and disrupting thyroid follicle structure. Interestingly, combinatorial treatment using BRAF and MEK inhibitors reversed the developmental effects induced by BRAFV600E and the authors identified a gene expression signature from zebrafish thyroid cancer. Gene expression studies identified TWIST2 as a crucial effector downstream of BRAF. The authors utilized CRISPR/Cas9 to genetically inactivate a TWIST2 orthologue, suppressing the effects of BRAFV600E and restoring thyroid morphology and hormone synthesis. This implies that TWIST2 expression is involved in an early stage of BRAFV600E-mediated transformation.

## Zebrafish to study TH transport defects

Once produced and released into the blood, THs require transport across the plasma membrane to gain access to the intracellular compartments. Monocarboxylate transporter 8 (MCT8) and 10 (MCT10) and the organic anion transporter proteins (OATPs) are all members of the solute carrier superfamily and have been shown to transport THs ^21^. In particular, MCT8 (also named SLC16A2) is necessary for the uptake of T3 in brain neurons, one of the most important cellular targets of T3 ^2^. The biological importance of MCT8 is proven by the manifestations of the Allan–Herndon–Dudley syndrome (AHDS) that carry dysfunctional mutations in the MCT8 gene. AHDS is an X-linked disease characterized by severe psychomotor retardation, muscle hypotonia and hypoplasia, ataxia, and impaired cognitive function. The underlying pathological mechanism is still elusive, and no cure for this condition is known ^2^. *Mct8*-knockout mice exhibit the same alterations in TH homeostasis as AHDS cases but without any brain dysfunction or locomotor disability ^22^. To improve understanding of AHDS, the zebrafish model has been extensively used in the last few years. Vatine and colleagues isolated the zebrafish *mct8* promoter, generating a transgenic line that confirms that *mct8* is primarily expressed in neurons, glial cells, and the vascular system, as in mammals ^23^. The first *mct8* model was generated 1 year later by the same group using MO-based gene knockdown ^24^. The *mct8* morphants exhibited impaired locomotor behavior and defective development of the cerebellum and mid-hindbrain boundary and apoptotic clusters in the zebrafish brain. Consistent with findings in patients, the authors also showed that the expression of zebrafish orthologues of the mammalian TSH, TH transporters, and deiodinases was affected in *mct8* morphants ^24^. More recently, they developed a mct8 zebrafish mutant model ( *mct8 ^–/–^*) using the zinc finger nuclease (ZFN)-mediated targeted gene editing system ^25^. Similar to patients, *mct8 ^–/–^* larvae displayed reduced locomotor activity and deficiency in behavioral performance, likely due to an altered development of sensory and motor neurons ^25^. Furthermore, *mct8 ^–/–^* mutants demonstrated neurological abnormalities and hypomyelination in the CNS. Pharmacological analysis showed that TH analogues and clemastine can partially rescue the hypomyelination in the CNS of *mct8 ^−/−^* larvae ^26^. Moreover, T3 treatment rescued hypomyelination in *mct8 ^−/−^* embryos before the maturation of the blood-brain barrier (BBB) but did not affect hypomyelination in later stages ^26^.

Taken together, these results emphasize the suitability of zebrafish as an animal model to study thyroid pathophysiology and AHDS by uncovering the effectiveness of early pharmacological treatments and BBB-targeted gene therapy that can enhance myelination in AHDS and possibly in other TH-dependent brain disorders ^27^.

## Zebrafish to study TH metabolism defects

Different tissues and cell types require TH at varying levels, and this is also the case throughout each of the developmental stages. Membrane transporters regulate TH entry into cells so as to deliver the proper intracellular hormone supply, and this is further fine-tuned by intracellular metabolism, which is controlled by three selenoprotein iodothyronine deiodinases (Ds). The only inheritable TH metabolism defect (THMD) known so far is the one resulting from recessive mutations in the selenocysteine insertion sequence-binding protein 2 (SECISBP2 [in short, SBP2]) gene, which influences selenoprotein synthesis, including the selenoenzymes deiodinases ^2^. The study of SBP2 defects in zebrafish is currently in progress in our lab (data not shown). There have been no reports as yet of humans with mutations in the deiodinase genes. Nevertheless, studies in animal models anticipated that mutations in other genes causing defective TH metabolism might have variable phenotypes.

Zebrafish studies indicated that D2 regulates the tempo of early embryonic development. D2-knockdown embryos, obtained by MO microinjection, resulted in a developmental delay, whereas D1 knockdown has minor effects ^28^. Houbrechts *et al*. have generated two different dio2 ( *dio2* ^–/–^) mutant zebrafish lines using ZFNs. Both mutants displayed a complete lack of D2 activity and a large diminution of T3 concentration in tissues ^29^. These mutants also exhibited early developmental perturbations, impaired locomotor activity, and long-term effects on growth and fertility ^29^. The D3 (encoded by two orthologues, *dio3a* and *dio3b*) was first implicated in the process of fin regeneration. Heijlen and colleagues recently showed that MO-mediated knockdown of either *dio3a* or *dio3b* results in partial loss of inner-ring deiodinase activity in the whole embryo, indicating that both paralogues may encode active enzymes ^30^. Moreover, the MO knockdown of both *dio3* transcripts induced a delay in development and reduced locomotor activity, suggesting that D3 knockdown can affect muscle and retinal development and function ^31, 32^. The same group also analyzed the transcriptome of the double knockdown D1/D2 (D1D2MO) and knockdown of D3 (D3MO) in zebrafish larvae. Both conditions resulted in a differential expression of genes involved in energy metabolism and muscle development ^31^. The up-regulation of transcripts encoding the ATP proteins seems to reflect a compensatory response to a decreased metabolic rate, a condition linked to hypothyroidism in D1D2MOs. Increased metabolic rate and stimulation of gluconeogenesis associated with delayed hatching and an increase in heart rate and carbohydrate content were reported in D3MO ^31^.

These findings provide new insight on the role of deiodinases during development, highlighting the importance of a correct TH balance during vertebrate development.

## Zebrafish to study TR defects

In zebrafish, TH action is mediated by different receptors (TRα1 and TRα2, TRβ1 and TRβ2) encoded by two genes ( *THRA* and *THRB*), with differing tissue distribution ^33^.

Until a few years ago, the term RTH was applied to the phenotype associated with dominant- negative (DN) mutations in the *THRB* gene. With the identification of DN mutations in the *THRA* gene, which presents with an extremely different phenotype, the syndromes are now identified as RTHβ and RTHα ^33^. The basic features of RTHβ include elevated serum free THs with non-suppressed TSH and goiter along with thyrotoxic and hypothyroid manifestations that are variable and largely unexplained (e.g. anxiety, tachycardia, failure to thrive, impaired hearing and color vision, attention-deficit hyperactivity disorder, and low bone density) in different tissues ^2^. We observed that the zebrafish *thrb* transcript is mainly expressed in the eye, pituitary, and otic vesicle of embryos ^34^. The generation of a DN model by MO microinjection demonstrated that TRβ isoforms are necessary for the proper development of sensory organs. Moreover, the TRβ morphants displayed the typical biochemical signature of RTHβ with a reduced sensitivity of the pituitary to L-T4 treatment ^34^. Suzuki and colleagues generated a TRβ2 fluorescent transgenic line and demonstrated that L-cone differentiation in the zebrafish retina requires TRβ2 activity ^35^. MO TRβ2 knockdown leads to L-cone reduction and a concomitant increase of UV cones. Conversely, ectopic expression of TRβ2 after cone differentiation produces cones with mixed opsin expression ^35^.

Since 2012, different heterozygous mutations in the *THRA* gene have been described in patients with RTHα ^4^. The associated symptoms are reminiscent of untreated CH (growth retardation, psycho-neuromotor disorders, delayed bone development, and bradycardia) but with a raised T3/T4 ratio and normal TSH levels. All variations fall in the ligand-binding domain (LBD), and mutant receptors present reduced T3 binding or defective interaction with corepressors or coactivators and variably affect the activity of the co-expressed normal receptors in a DN manner. As a consequence, RTHα patients present variable responses to TH treatment ^4^. During zebrafish embryonic development, TRα transcript is mainly localized in the CNS, heart, gastrointestinal tract, and pronephric ducts. Moreover, embryos harboring DN mutations in the LBD of zebrafish TRα isoforms recapitulated most of the key clinical and biochemical manifestations (e.g. growth and brain defects, delayed cartilage development, defective heart function, and high T3/T4 ratio) described in RTHα patients ^34^. We observed increased pituitary *dio2* and reduced *dio3a* and *dio3b* that can explain the peculiar biochemical features found in patients with RTHα. We also reported that zebrafish and human TRs were functionally interchangeable and that high T3 doses partially reverted the DN action of mutant hTRα in morphants ^34^.

More recently, we established a simplified RTHα *in vivo* model by the direct microinjection of human *THRA* transcripts into zebrafish eggs ^36^. The embryos injected with human variants (hTRα-injected embryos) undergo profound morphological, neurological, cardiovascular, hematological, skeletal, and biochemical alterations reminiscent of RTHα in humans. Consistently with *in vitro* results, an efficient rescue of the disease phenotypes was always seen upon the addition of a high T3 concentration in the harvesting water only in embryos injected with the missense variants, which are partially resistant to T3 in contrast with the complete refractoriness of the truncated mutations ^36^. Therefore, the whole of these findings support the view that the observed fish phenotypes are specifically caused by the DN activity exerted by hTRα mutants on zTRs and that zebrafish may become a useful ‘biotool’ to test new therapeutic approaches to cure RTHα. Indeed, treatment with a TH analogue selectively targeting mutant TRα may be preferable to thyroxine treatment, which can have untoward effects on TRβ-mediated pathways.

While this review was under revision, the Stainier lab described an important role for TH action during pancreatic islet maturation using a novel TH-responsive reporter line ^37^. They found that both α and β cells become targets of endogenous TH signaling during the larval-to-juvenile transition. Therefore, it appears that endogenous THs, beyond their requirement for the embryo-to-larval transition described by Liu and Chan several years ago ^38^, are also required during the larval transition for the functional maturation of α and β cells in order to maintain glucose homeostasis.

## Zebrafish to study the toxic effects on thyroid function

A number of studies have been finally reported in recent years on the use of zebrafish to study the effects of endocrine disruptor chemicals (EDCs). Several evaluated the effects of bisphenol AF or S (BPAF or BPS) exposure ^39– 41^. Treatment with such pollutants induced variable changes in TH concentrations as well as in TH metabolism and action. These data provide interesting insights on the toxicity of these compounds but are often difficult to interpret, as opposite effects (decrease or increase of thyroxine concentrations) are seen in different studies ^39, 40^ and significant effects with contrasting or divergent results on gene expression are seen in both thyroid and hypothalamic-pituitary tissues.

## Conclusions

In this review, we attempted to summarize the most recent findings concerning thyroid development and TH action that have been obtained in zebrafish during the last four years ( Figure 1). All of these data highlight the great potential of this model to investigate thyroid diseases. Zebrafish embryos are particularly useful thanks to their small size, rapid development, and optical transparency. Given the conservation of the molecular mechanisms regulating thyroid development and TH action in zebrafish and mammals, the availability of fluorescent reporters and several techniques for genetic manipulation make zebrafish a powerful model to investigate the mechanisms underlying human developmental or neoplastic diseases. The generation of specific genetic models can efficiently harness the advantages of zebrafish embryos for a phenotypic characterization of novel candidate genes. In addition, these models will also be suitable for drug screening and will greatly accelerate the development of new treatment protocols.
